# The influence of age, anthropometric and metabolic variables on LDI_FLARE_ and corneal confocal microscopy in healthy individuals

**DOI:** 10.1371/journal.pone.0193452

**Published:** 2018-03-08

**Authors:** Sanjeev Sharma, Victoria Tobin, Prashanth R. J. Vas, Rayaz A. Malik, Gerry Rayman

**Affiliations:** 1 Diabetes Research Unit, The Ipswich Hospital NHS Trust, Ipswich, United Kingdom; 2 Department of Diabetes, The Kings College NHS Foundation Trust, London, United Kingdom; 3 Weill Cornell Medicine-Qatar, Doha, Qatar & Institute of Cardiovascular Medicine, University of Manchester, Manchester, United Kingdom; Bascom Palmer Eye Institute, UNITED STATES

## Abstract

**Introduction:**

The laser Doppler imaging (LDI) _FLARE_ and corneal confocal microscopy (CCM) are reliable markers of small fibre function (SFF) and structure (SFS), respectively, but the impact of potential confounding variables needs to be defined. The objective of this study was to determine the influence of age, anthropometric and biochemical variables on LDI and CCM.

**Methods:**

80 healthy volunteers (43 males) (age: 39.7±15.2 yrs.) underwent LDI_FLARE_ and CCM assessment and the effect of age, anthropometric and biochemical variables was determined using multivariate analysis.

**Results:**

There was an age-related decline in LDI_FLARE_ (0.07cm^2^/yr; R^2^ = 0.669; p = <0.0001) and CCM parameters (CNFD: 0.05 fibres/mm^2^ /yr; R^2^ = 0.590; p = <0.0001, CNBD: 0.06 branches/mm^2^/yr; R^2^ = 0.549; p = 0.001and CNFL 0.07 mm/mm^2^/yr; R^2^ = 0.369; p = 0.009). BMI did not influence SFF (p = 0.08) but had a significant independent association with CNFD (p = 0.01). Fasting triglycerides (TG) independently influenced the LDI_FLARE_ (β_c_:-0.204; p = 0.008) and all CCM indices (β_c_:-0.191 to -0.243; p = <0.05). HbA_1c_ was significantly associated with CNFD only (p = 0.001) but not with LDI_FLARE_, CNBD or CNFL (p = ≥0.05). Blood pressure and total cholesterol were not associated with LDI_FLARE_ or any CCM parameters. There was a significant correlation between LDI_FLARE_ and all CCM parameters (p = ≤0.01).

**Conclusions:**

This study shows that in healthy controls, both SFF measured by LDI_FLARE_ and SFS assessed by CCM showed a significant inverse correlation with age and triglycerides, perhaps suggesting the use of age-specific normative values when interpreting these outcomes. Furthermore, this study shows that in healthy controls, despite measuring different neural parameters, both methods correlated significantly with each other.

## Introduction

Diabetic polyneuropathy (DPN) is the commonest chronic complication of diabetes mellitus with a prevalence of up to 50% [1]. Prospective studies in both type-1 (TIDM) and type-2 (T2DM) diabetes have shown that the prevalence of DPN increases with the duration of diabetes [2–4] and is associated with significant morbidity and mortality and health economic cost [5].

There is an increasing drive for the earlier detection of DPN by quantifying large myelinated Aα and Aβ and in particular Aδ and C fibres [6, 7]. Traditionally, neuropathic symptoms and signs along with nerve conduction studies were used to diagnose DPN and assess therapeutic outcome [8]. However, there is now increasing evidence to suggest that small fibre dysfunction and damage may precede large fibre neuropathy in subjects with impaired glucose tolerance [9, 10] and diabetes [11, 12] and predicts future-incident DPN [13]. This has led to the development of newer non-invasive techniques to assess small nerve fibre integrity.

Small nerve fibre integrity can be evaluated by quantifying small fibre structure (SFS) and function (SFF). SFS can be evaluated via skin biopsy by measuring IENFD and in the cornea using corneal confocal microscopy (CCM). SFF can be evaluated using the laser Doppler imager flare technique (LDI_FLARE_), quantitative sensory testing (QST) of warm and cold thermal thresholds and the quantitative sudomotor axon reflex test (QSART) [14]. However, IEFND is invasive and requires considerable expertise for accurate quantification and QST is relatively time consuming, subjective and have a high coefficient of variation [15]. QSART remains limited to research centers due to the technical complexity, and again is a relatively time consuming technique [16].

CCM is a rapid, non-invasive ophthalmic technique that can accurately quantify corneal nerve morphology in diabetic patients with minimal and more advanced neuropathy [17], correlates with IENFD [18] and can be quantified objectively and reproducibly using automated algorithms [19, 20]. The LDI_FLARE_ method has also been shown to be a sensitive method for the detection of early small fibre dysfunction and also correlates with dermal nerve fibre density [21]. Furthermore, we have shown that the flare size is influenced by long term glycaemia in T1DM whilst features of the metabolic syndrome appear more important determinants in IGT and T2DM [22, 23].

Given the potential utility of CCM and LDI_FLARE_ in clinical screening and early identification of DPN, it is important to identify variables which may influence these measurements in a healthy population. Whilst corneal sensitivity may decline with age [24, 25], corneal nerve fibre density has been related in some [26–28] but not all [29–31] studies. There are a limited number of studies showing age-dependent deterioration of both the LDI_FLARE_ and thermal threshold testing [32, 33].

This is the first study to evaluate the effect of age and other clinical variables on both small fibre function and structure in a cohort of healthy controls.

## Materials and methods

### Ethical approval

This study was conducted in accordance with the Declaration of Helsinki and approved by the ethics committee of the NRES Committee East of England—Norfolk, UK. (REC reference: 13/EE/0162). All subjects provided written informed consent.

### Study design and selection of participants

Eighty (80) healthy subjects, aged 18 to 80 years, were recruited by invitation. This study is part of a larger longitudinal neuropathy study currently in progress in our centre involving both healthy subjects alongwith Type-1 and Type-2 diabetes patients. The current data presented was obtained as part of this larger study between 2015–2017. Participants were unselected from the local population including hospital staff where the ethnicity is 97% Caucasian.. Subjects with a history of impaired fasting glycaemia, pre-diabetes or diabetes (type-1, type-2 or other specific types) as per diagnostic criteria of the American Diabetes Association, 2017, were excluded [34] based on a fasting blood glucose (FBG) and glycosylated haemoglobinA_1c_ (HbA_1c_). Subjects were also excluded if there was any history of hypertriglyceridaemia, alcohol abuse, vitamin B12 and folate deficiency, thyroid or connective tissue disorders, renal or hepatic failure, malignancy, inherited neuropathy or exposure to any toxins including chemotherapy.

### Clinical and biochemical assessment

All participants underwent testing for fasting glucose HbA_1c_, lipids, thyroid, renal and hepatic profiles, vitamin B12 and folate. Overall, five patients—three due to pre-diabetes and two due to subclinical hypothyroidism were excluded. All screened patients underwent detailed neurological examination with the modified Neuropathy Disability Score (NDS) [35]. Vibration perception threshold was measured on the toes using the Neurothesiometer (Horwell Scientific, UK) and expressed as millivolts (mV). Sural nerve conduction velocity (SNCV) and amplitude (SNAP) were assessed in one leg (NC-stat|DPNCheck system (Neurometrix, Waltham, MA).

### Corneal confocal microscopy (Fig 1)

**Fig 1:**
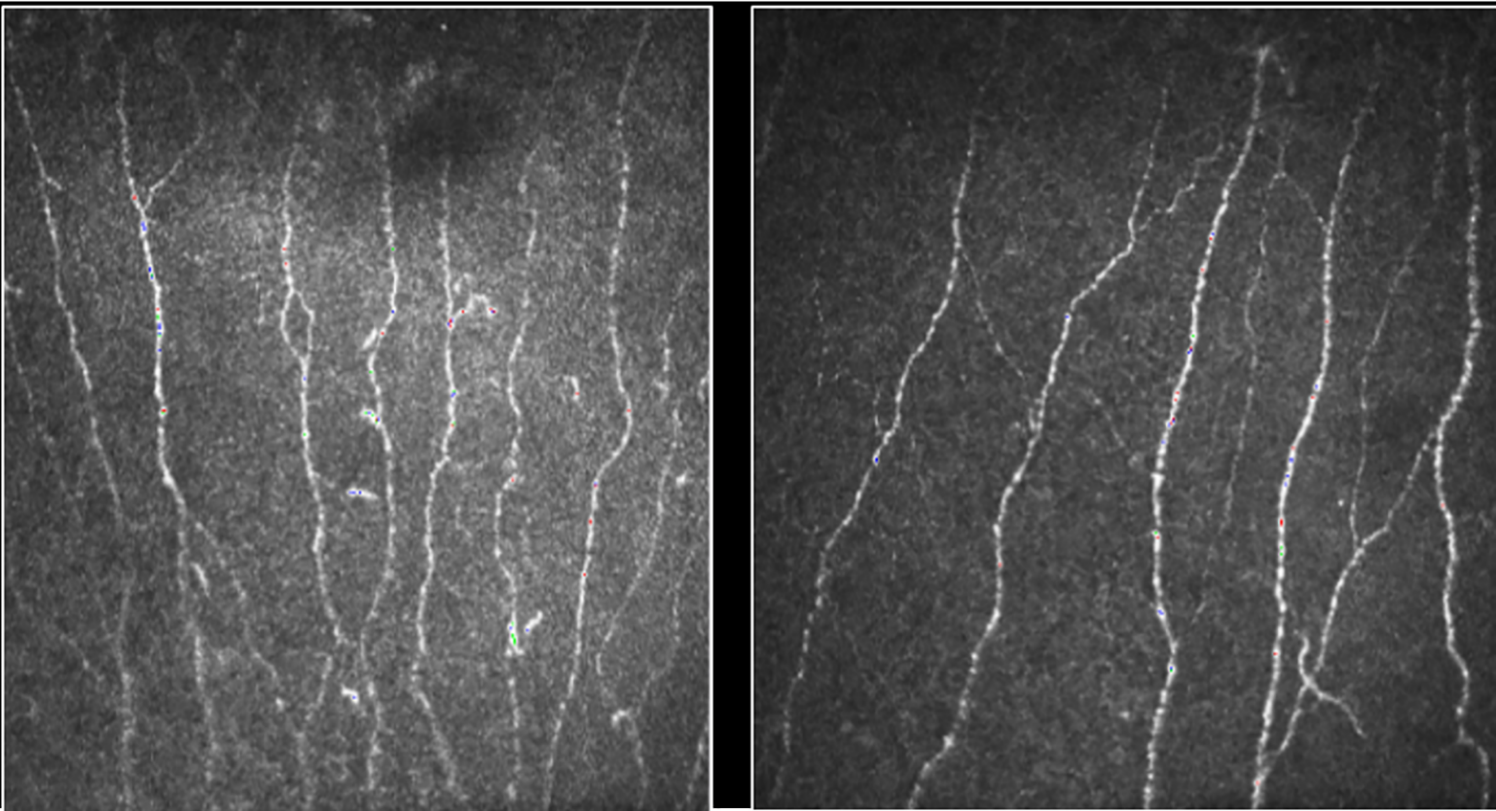
CCM images at original image x 700 times magnification. The figure on the left is from a 25-year old HC with CNFD at 61.89 no./mm^2^ (>75^th^ centile) while on the right is from a 71-year old HC with CNFD at 40.12/mm^2^ (<25^th^ centile).

CCM was performed using the laser scanning Heidelberg Retina Tomograph III confocal microscope with Rostock Corneal Module (Heidelberg Engineering GmbH, Dossenheim, Germany). Prior to scanning, topical anaesthesia (Minims^®^: Oxybuprocaine hydrochloride 0.4%; Bausch & Lomb, Kingston upon Thames, Surrey, UK) was applied to the cornea of both eyes followed by topical application of a viscous gel (Viscotears^®^: Carbomer 980 polyacrylic acid 0.2%; Alcon Eye Care, Frimley, Surrey, UK) to form a aqueous medium between the applanated corneal surface and disposable TomoCap covering the objective lens. Images were obtained from both eyes using our established methodology [36, 37] with a total duration for examination of both eyes between five to ten minutes. Six best images from both eyes were selected manually and automated image analysis was performed using purpose-written, proprietary software (CCM Image Analysis tool v1.1: Imaging Science and Biomedical Engineering, University of Manchester, Manchester, UK)[19]. The specific parameters measured per frame were: CNFD (number of fibres/mm^2^), CNBD (number of fibres / mm^2^), and CNFL (length in mm/ mm^2^) [36].

### LDI_FLARE_ (Fig 2)

**Fig 2:**
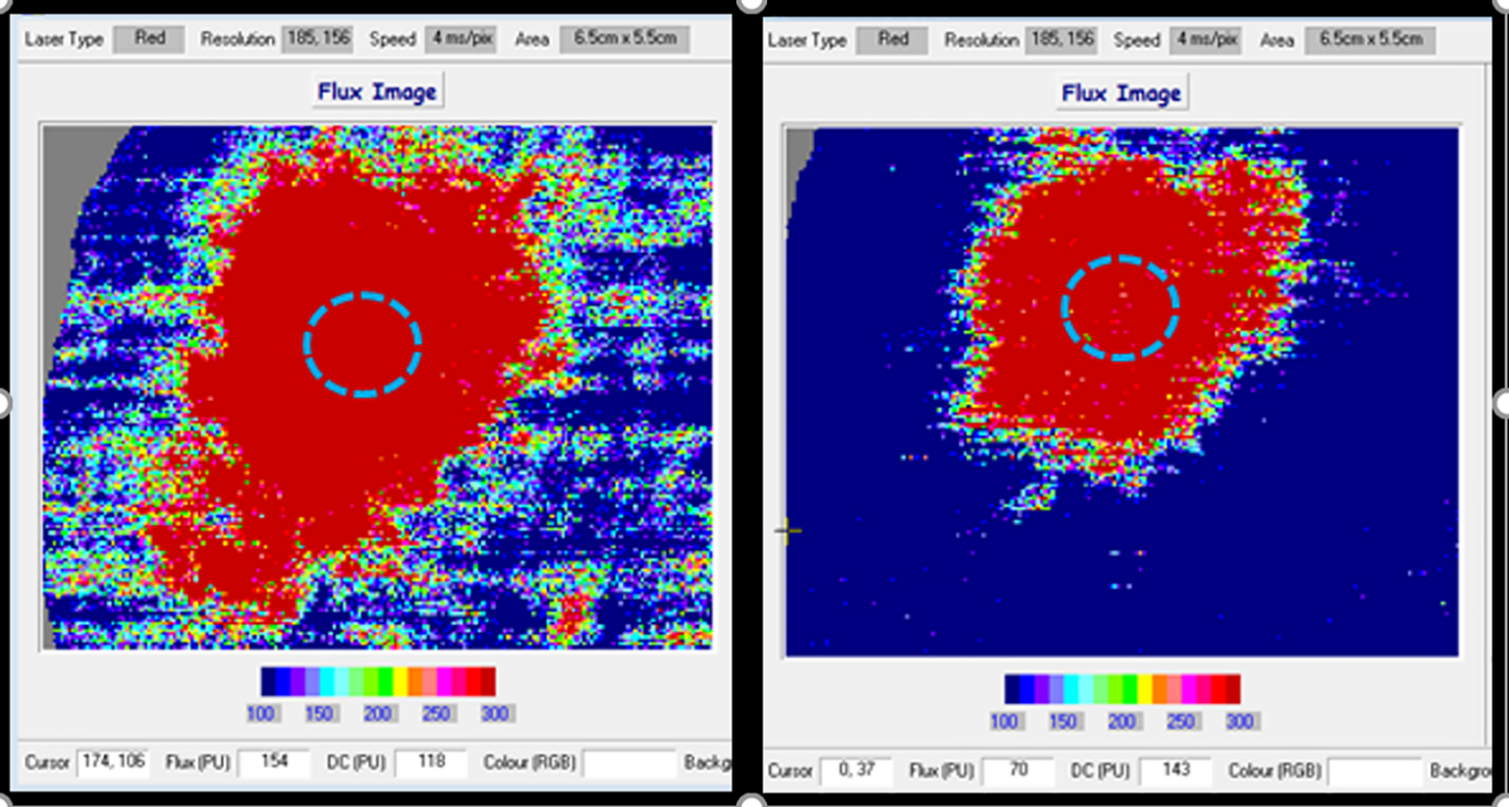
LDI_FLARE_ images. The image on the left is from a 22-year old HC with LDI_FLARE_ at 12.44 cm^2^ (>75^th^ centile) while image on the right is from a 75-year old HC with LDI_FLARE_ at 5.17 cm^2^ (<25^th^ centile).

LDI_FLARE_ was undertaken using our established technique [38]. The procedure is carried out in a temperature-controlled room (25 ± 1°C) after acclimatization [38]. A 1 cm^2^ heating probe was applied to the dorsum of the foot, 2-3cm proximal to the first inter-phalangeal space and the skin was sequentially heated for 6 minutes in a step-wise algorithm: 44°C for 2 minutes, 46°C for 1minute, and 47°C for 3 minutes and the reference area was scanned using a laser Doppler imager (Moor Instruments, Axminster, UK). On the flux image, the area of the LDI_FLARE_ was outlined using the free drawing tool and the results were expressed in cm^2^ and measured using the Moor V 5.3 software.

### Calculation of rate of decline of small fibre structure and function with age

The age range in our cohort of HC was between 19 and 74 years and was normally distributed. Using the age-related reference intervals suggested by Wright et al [39], we segregated our HC into four age groups (<30 years, 30–44, 45–59 and >60 years). The percentage difference between the 50th centile of the youngest and oldest group was divided by a factor of 6, covering the six decades, to establish the percentage rate of decline per decade using the formula:

$$\frac{\left[1-\{(Median\;flare\;area\;in\;Oldest\;group)/(Median\;flare\;area\;in\;youngest\;group)\}x100\right]}{Number\;of\;decades\;in\;years}$$

### Statistical analysis

Statistical analysis was performed using SPSS (version 20 for Windows) and StatsDirect version 3. Clinical characteristics as quantitative variables are expressed as mean ± standard deviation. Normal distribution of the data was determined with the Kolmogorov-Smirnov test. To examine independent effect of age, BMI, HbA_1c_ and lipid indices on both LDI_FLARE_ and CCM parameters, multivariate linear regression was used. Pearson’s bivariate correlation was used, to assess the association of small fibre function and structure large fibre methods VPT, SNAP, SNCV) and to examine the relationship between LDI_FLARE_ and CCM outcomes. The co-efficient of variation for CCM and LDI_flare_ is 7.8% and 8.7%, respectively.

## Results

### Relationship between anthropometric variables and small fibre function and structure

80 gender balanced (42 males; 52.5%; p = 0.45) subjects with a wide age range of 39.68±15.17 years (19–74 years) were studied. Age, gender, body mass index (BMI), lipid indices and systolic and diastolic blood pressure are shown in Table 1. Multivariate regression analysis showed that increasing age influenced LDI_FLARE_ (p = <0.0001; beta coefficient (βc):-0.728) and all three CCM parameters: CNFD (p = <0.0001; βc:-0.627), CNBD (p = 0.001; βc:-0.741) and CNFL (p = 0.009; βc:-0.508). There was a weak but significant association between BMI and CNFD. Gender and both systolic and diastolic blood pressures did not correlate with any of the small fibre assessments (p = ≥0.05).

**Table 1:** Clinical characteristics of study population and independent effect of age, BMI, HbA_1c_ and lipid indices on both LDI_FLARE_ and CCM parameters using multivariate linear regression. Bivariate correlation was used to examine the relationship between the LDI_FLARE_, CCM outcomes and large fibre methods used (VPT, SNCV and SNAP).

Characteristics		Correlations*			
		LDI_FLARE_	CCM		
			CNFD	CNBD	CNFL
**Age** *(years)*	39.68±15.17	***p = <0*.*0001*** (-0.728)	***p = <0*.*0001*** (-0.627)	***p = 0*.*001*** (-0.741)	***p = 0*.*009*** (-0.508)
**BMI** *(kg/ m*^*2*^*)*	29.09±19.16	*p = 0*.*56* (-0.108)	***p = 0*.*03* (**-0.318)	*p = 0*.*23* (-0.114)	*p = 0*.*27* (-0.121)
**SBP** *(mm Hg)*	132±21	*p = 0*.*81* (-0.045)	*p = 0*.*77* (-0.067)	*p = 0*.*67* (-0.065)	*p = 0*.*80* (-0.049)
**DBP** *(mm Hg)*	76±13	*p = 0*.*11* (-0.109)	*p = 0*.*16* (-0.098)	*p = 0*.*19* (-0.095)	*p = 0*.*10* (-0.105)
**FBG** *(mmol/L)*	4.91±0.49	***p = 0*.*009*** (-0.291)	*p = 0*.*07* (-0.121)	***p = 0*.*001*** (-0.375)	*p = 09* (-0.101)
**HbA**_**1c**_ *(%)*	4.86±.0.36	*p = 0*.*06* (-0.145)	***p = 0*.*001*** (-0.375)	*p = 0*.*06* (-0.167)	*p = 0*.*08* (-0.179)
**Triglyceride** *(mmol/L)*	1.89±0.56	***p = 0*.*008*** (-0.204)	***p = 0*.*004*** (-0.232)	***p = 0*.*011*** (-0.243)	***p = 0*.*04*** (-0.191)
**Total Cholesterol** *(mmol/L)*	4.50±0.85	*p = 0*.*45* (-0.088)	*p = 0*.*41* (-0.076)	*p = 0*.*50* (-0.0.75)	*p = 0*.*55* (-0.056)
**LDI**_**FLARE**_ (cm^2^)	9.11±2.17	-	***p = <0*.*0001*** (0.75)	***p = 0*.*01*** (0.46)	***p = 0*.*001*** (0.59)
**VPT** (volts)	6.01±3.75	*p = 0*.*11* (0.12)	*p = 0*.*13* (0.13)	*p = 0*.*23* (0.09)	***p = 0*.*04*** (0.30)
**SNCV** (m/s)	50.23±5.69	***p = <0*.*0001*** (0.81)	***p = 0*.*01*** (0.44)	*p = 0*.*06* (0.29)	*p = 0*.*19* (0.10)
**SNAP** (μV)	18.49±4.13	***p = <0*.*0001*** (0.77)	***p = 0*.*009***	*p = 0*.*08* (0.19)	*p = 0*.*22* (0.11)

Variables expressed as Mean ± SD. BMI: body mass index; SBP: systolic blood pressure; DBP: diastolic blood pressure; FBG: fasting blood glucose; HbA_1c_: glycosylated haemoglobin A_1c;_ LDI_FLARE_: laser Doppler imager flare; CNFD: corneal nerve fibre density; CNBD: corneal nerve branch density; CNFL: corneal nerve fibre length; VPT: vibration perception threshold; SNCV: sural nerve conduction velocity; SNAP: sural nerve amplitude;

* βc: beta coefficient where multivariate linear regression used; R^2^: Pearson’s correlation of co-efficient of determination). ***Significance = p<0*.*05***.

### Relationship between biochemical variables and small fibre function and structure (after multivariate adjustment)

FBG influenced LDI_FLARE_ (p = 0.009; βc:-0.291) and CNBD (p = 0.001; βc:-0.375) but not CNFD (p = 0.76) or CNFL (p = 0.13). HbA_1c_ influenced CNFD (p = 0.001; βc:-0.375) but not CNBD, CNFL or LDI_FLARE_, (p = ≥0.05). There was a significant independent correlation between TG and LDI_FLARE_ (p = 0.008; βc:-0.204), CNFD (p = 0.004; βc:-0.232), CNBD (p = 0.011; βc:-0.243) and CNFL (p = 0.04; βc:-0.191).

### Relationship between small fibre function and structure

LDI_FLARE_ correlated with all CCM parameters (p = ≤0.01; R^2^ = 0.46–0.75) (Table 1).

### Relationship with measures of large fibre dysfunction

There was significant correlation between SNCV and SNAP with LDI_FLARE_ (both p = <0.0001) and CNFD (p = 0.01 and p = 0.009 respectively) and weakly between VPT and CNFL (p = 0.04) (Table 1). VPT (p = 0.001), SNCV (p = 0.002) and SNAP (p = 0.001) were significantly correlated with age; however did not correlate with gender, BMI, blood pressure, FPG, HbA1c or lipid profiles (p = ≥0.05; data individually not presented).

### Rate of decline in small fibre function and structure

**Fig 3:**
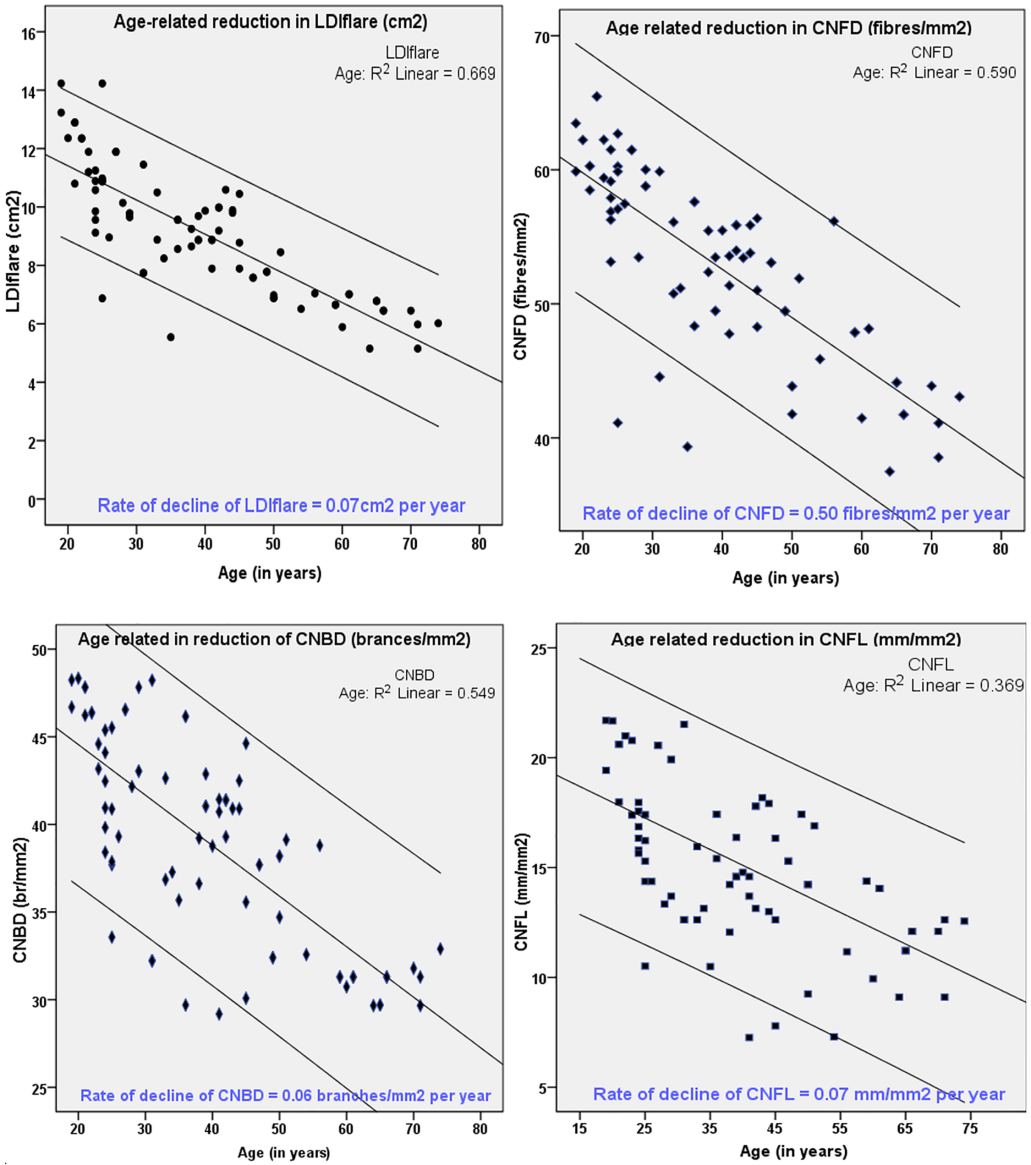
Relationship between age and both LDI_FLARE_ and CCM parameters. The lines above and below the trend line describes the 95th and 5th centiles respectively.

The distribution centiles for the four age categories are shown in Table 2. The median (50^th^) centile of the youngest and oldest group was used to derive the rate of decline. Fig 3 shows that the rate of decline of the LDI_FLARE_ was 0.07cm^2^/year (p = <0.0001; βc: -0.728); CNFD was 0.05 fibres/mm^2^ per year (p = <0.0001; βc: -0.627), CNBD was 0.06 branches/mm^2^ per year (p = 0.001; βc: -0.741) and CNFL was 0.07 mm/mm^2^ per year (p = 0.009; βc: -0.508).

**Table 2:** Centile charts derived from 80 healthy controls divided into 4 age groups.

Assessment	Centiles	<30 years	30–44 years	45–59 years	>60 years
*Number of subjects in each group*		*28*	*25*	*15*	*12*
**LDI**_**FLARE**_ (cm^2^)	**5**^**th**^ **centile**	7.81	6.20	6.15	5.15
	**25**^**th**^ **centile**	9.80	8.61	6.88	5.91
	**50**^**th**^ **centile (median)**	10.94	9.20	7.58	6.45
	**75**^**th**^ **centile**	12.35	9.88	7.89	6.78
	**95**^**th**^ **centile**	14.23	11.19	9.45	7.01
**CNFD** (no/ mm^2^)	**5**^**th**^ **centile**	46.53	40.90	41.77	37.49
	**25**^**th**^ **centile**	57.58	49.48	45.87	41.19
	**50**^**th**^ **centile (median)**	59.87	53.47	49.45	42.40
	**75**^**th**^ **centile**	61.50	55.87	53.08	44.14
	**95**^**th**^ **centile**	65.47	59.19	51.13	47.24
**CNBD** (no/ mm^2^)	**5**^**th**^ **centile**	35.42	33.34	30.08	29.66
	**25**^**th**^ **centile**	40.89	36.74	32.40	28.70
	**50**^**th**^ **centile (median)**	44.34	40.90	35.57	28.33
	**75**^**th**^ **centile**	46.54	41.97	38.19	26.77
	**95**^**th**^ **centile**	48.29	47.60	35.45	26.01
**CNFL** (mm/mm^2^)	**5**^**th**^ **centile**	11.79	8.23	7.30	7.10
	**25**^**th**^ **centile**	15.68	12.80	11.17	10.26
	**50**^**th**^ **centile (median)**	17.77	14.59	14.38	10.10
	**75**^**th**^ **centile**	20.60	17.42	16.34	9.78
	**95**^**th**^ **centile**	21.69	20.52	13.11	9.09

## Discussion

The techniques of CCM and LDI_FLARE_ allow rapid, non-invasive detection of early small fibre deficits in DPN [11]. This study demonstrates that age has a highly significant independent effect on both LDI_FLARE_ and CCM measures, urging the need to establish age-stratified thresholds when these techniques are deployed for the diagnosis of DPN [40].

Previously age has been shown to influence intraepidermal nerve fibre density in some [41, 42], but not other [43] studies. The influence of anthropometry and biochemical variables has not been examined in healthy subjects. Similarly, for corneal nerve fibre density, Erie et al [29], Marfurt et al [30] and Patel et al [31] found no significant association with age, whilst Grupcheva et al found an independent age-related reduction in corneal nerve density [26]. Neiderer et al found an age-related decrease of sub-basal corneal nerve density of 0.9% per year [27]. In a 3-year prospective study of 49 healthy individuals Dehgani et al reported a significant linear decrease of 0.05 mm/mm^2^ in CNFL per one year increase in age [44], which is comparable to the decrease observed in our study. In a recent multicentre collaborative study there was a significant linear age-dependent decrease in CNFD (-0.164/mm^2^ per year for men, and -0.161/mm^2^ per year for women; p = <0.0001)) and CNFL (R^2^ = 0.026; p = 0.003),but no change in CNBD; height, weight, and BMI had no influence [28]. Lin et al [33] have commented that age is the most significant factor in determining sensory thresholds compared with the other factors of gender and anthropometric parameters and similarly, Hafner et al [45]have shown that thermal threshold detection increases with age. We have previously demonstrated a significant age-dependent decline in LDI_FLARE_ size (r = -0.42; p = <0.0001) of 0.056 cm^2^ per year, giving a percentage loss of 5.5% per decade.

The present study found no influence of gender, BMI or blood pressure on both small fibre function and structure in keeping with other studies of CCM and the LDI_FLARE_ in HC. [28, 46, 47]. The effects of glycaemic measures on CCM and the LDI_FLARE_ were inconsistent; HbA_1c_ showed a significant inverse correlation with CNFD but not with the LDI_FLARE_. In contrast, FBG correlated significantly with LDI_FLARE_ and CNBD but not with other CCM parameters. Other studies of CCM in healthy controls also show conflicting results. A recent multinational data set report by Tavakoli et al [28] did not find any association between CCM and HbA_1c_ although FBG was not done; however in another study by Wu et al [40], HbA_1c_ was show to be the only independent clinical factor to account for variations in corneal nerve fibre length, independent of age. We did not expect an association between FBG and the LDI_FLARE_; our previous study with a different cohort of HC did not demonstrate any association of HBA_1c_ or FBG with the LDI_FLARE_ but CCM was not performed in this study. Further studies including longitudinal studies will be necessary to determine whether the association between fasting glucose and the LDI_FLARE_ is real or a chance statistical finding.

Both the LDI_FLARE_ and CCM correlated significantly with fasting triglycerides, but not total cholesterol. The association of TG with diabetic neuropathy has been previously highlighted in both observational [48–50] and interventional [51–53] studies. Indeed we have previously reported an inverse correlation between LDI_FLARE_ and TG in normoglycaemic individuals [46]. To our knowledge, the present study is the first to find a relationship between TG and both SFF and SFS in healthy controls.

An intriguing finding of this study is that all three CCM indices (CNFD, CNBD and CNFL) correlated significantly with the LDI_FLARE_ despite examining anatomically very different areas and tissues. This suggests that these measures are influenced by common determinants of neural health and could be used interchangeably in healthy controls. However, whether these are affected in the same manner in people with diabetes needs to be determined before suggesting that they can be used interchangeably to estimate early small fibre diabetic neuropathy; additionally, the effect of variables such as glycaemic control, TG, hypertension, other metabolic and inflammatory markers will need to be better defined. This will require high quality longitudinal studies. Additional measures of small fibre structure (IENFD) and function (QST) may provide a more robust and comprehensive analysis.

Another limitation of this study is that the participants were healthy volunteers; therefore, this study cannot be considered to be a true population-based study. Hence, it is not powered to study the effect of other demographic factors like geographical location including altitude, lifestyle choices (e.g. athletes’ vs sedentary living), dietary patterns and smoking history. Whilst these are important factors which might further define the utility of these methods in the early detection of DPN, it will require much larger multicentred studies in diverse populations to understand their possible roles.

To conclude, this large cross-sectional study confirms the importance of age when interpreting LDI_FLARE_ and CCM to diagnose small fibre neuropathy. Furthermore, it identifies the influence of glycaemia and in particular triglycerides on both small fibre structure and function, even in healthy individuals. Finally, the significant correlation between and CCM in healthy subjects validates their use in studies evaluating small fibre neuropathy.

## Supporting information

S1 File Anonymised data set. (XLSX)

## Abbreviations

CCM: Corneal Confocal Microscopy
CNBD: Corneal Nerve Branch Density
CNFD: Corneal Nerve Fibre Density
CNFL: Corneal Nerve Fibre Length
DPN: Diabetic Polyneuropathy
FPG: Fasting Plasma Glucose
HbA_1c_: Glycated Haemoglobin A_1c_
HC: Healthy Controls
IENFD: Intra-Epidermal Nerve Fibre Density
LDI_FLARE_: Laser Doppler Imaging flare
NCS: Nerve Conduction Studies
NCV: Nerve Conduction Velocity
QST: Quantitative Sensory Tests
SNAP: Sural Nerve Amplitude
SNCV: Sural Nerve Conduction Velocity
T1DM: Type-1 Diabetes Mellitus
T2DM: Type-2 Diabetes Mellitus
TC: Total Cholesterol
TG: Triglyceride
VPT: Vibration Perception Threshold
